# Lipid accumulation product: a simple and accurate index for predicting metabolic syndrome in Taiwanese people aged 50 and over

**DOI:** 10.1186/1471-2261-12-78

**Published:** 2012-09-24

**Authors:** Jui-Kun Chiang, Malcolm Koo

**Affiliations:** 1 Department of Family Medicine, Buddhist Dalin Tzu Chi General Hospital, Chiayi, Taiwan; 2 Department of Biotechnology, Chia Nan University of Pharmacy & Science, Tainan, Taiwan; 3 Dalla Lana School of Public Health, University of Toronto, 155 College Street, Toronto, ON M5T 3M7, Canada

**Keywords:** Adiposity, Chinese, Metabolic syndrome, Prediction

## Abstract

**Background:**

Lipid accumulation product (LAP) has been advocated as a simple clinical indicator of metabolic syndrome (MS). However, no studies have evaluated the accuracy of LAP in predicting MS in Taiwanese adults. The aim of our investigation was to use LAP to predict MS in Taiwanese adults.

**Methods:**

Taiwanese adults aged 50 years and over (*n* = 513) were recruited from a physical examination center at a regional hospital in southern Taiwan. MS was defined according to the MS criteria for Taiwanese people. LAP was calculated as (waist circumference [cm] − 65) × (triglyceride concentration [mM]) for men, and (waist circumference [cm] − 58) × (triglyceride concentration [mM]) for women. Simple logistic regression and receiver-operating characteristic (ROC) analyses were conducted.

**Results:**

The prevalence of MS was 19.5 and 21.5% for males and females, respectively. LAP showed the highest prediction accuracy among adiposity measures with an area under the ROC curve (AUC) of 0.901. This was significantly higher than the adiposity measure of waist-to-height ratio (AUC = 0.813).

**Conclusions:**

LAP was a simple and accurate predictor of MS in Taiwanese people aged 50 years and over. LAP had significantly higher predictability than other adiposity measures tested.

## Background

Metabolic syndrome (MS) is a condition that includes the presence of a cluster of risk factors specific for cardiovascular disease [1,2], type 2 diabetes mellitus [3], hypertension [4], and all-cause mortality [5]. Several diagnostic criteria have evolved since the World Health Organization task force on diabetes identified insulin resistance as the dominant cause of MS in 1998 [6]. Over time, several definitions of MS have been proposed, with criteria based on various combinations of abdominal or visceral obesity, insulin resistance, raised blood pressure, and dyslipidemia [7]. In Taiwan, the latest definition of MS (MS-TW) recommended by the Bureau of Health Promotion in the Department of Health is based on the United States National Cholesterol Education Program Adult Treatment Panel III criteria (NCEP ATP III) [8], with modifications. According to MS-TW, the diagnosis of MS is made when at least three of the following five risk determinants are present: waist circumference ≥ 90 cm in men and ≥ 80 cm in women; blood pressure > 130/85 mmHg or patient is taking antihypertensive medications; high-density lipoprotein cholesterol (HDL-C) < 0.9 mM in men and < 1.03 mM in women; fasting plasma glucose ≥ 5.6 mM or patient is undergoing regular treatment for diabetes mellitus; and triglyceride level ≥ 1.70 mM. Nevertheless, it would be useful if a simpler index is available for easy diagnosis of individuals at risk of MS in clinical settings.

Recently, Kahn proposed the use of lipid accumulation product (LAP), a novel index of central lipid accumulation, to predict the risk of MS [9]. LAP is based on a combination of waist circumference and plasma triglyceride levels. The LAP method was shown to predict diabetes [10] and recognize cardiovascular risk [11] better than body mass index (BMI) in previous studies. LAP was also associated with all-cause mortality in non-diabetic patients at high cardiovascular risk [12] and all-cause, cardiovascular, and congestive heart failure mortality in postmenopausal women [13]. In addition, LAP has been tested in the Chinese population for predicting diabetes. Analysis of areas under the curves (AUCs) for receiver-operating characteristic (ROC) curves indicated that LAP was able to predict diabetes better than waist-to-hip ratio, waist circumference, and BMI, in both men and women [14]. LAP has also been applied to healthy Argentinian men, with an AUC of 0.91 observed for MS [15]. With Taiwanese people, we conducted a cross-sectional study to assess the accuracy of LAP employing a number of adiposity measures. The adiposity measures included BMI, waist circumference, hip circumference, waist-to-hip ratio, and waist-to-height ratio, central obesity (defined as waist circumference ≥ 90 cm in males and ≥ 80 cm in females), and body adiposity index [8].

## Methods

### Study subjects

Study subjects were individuals recruited from a physical examination center at a regional hospital in southern Taiwan between May 2007 and April 2008. The study was approved by the Institutional Review Board of the hospital, and written informed consent was provided by all participants before enrollment.

### Demographic information and clinical measurements

Demographic information including age, sex, body weight, height, waist circumference, and hip circumference of the subjects was recorded. Waist circumference was measured at the umbilicus (with thin clothes worn), and hip circumference measured around the widest portion of the buttocks [16]. Subjects who were below 50 years or on lipid-lowering medications were excluded from the study. BMI was calculated by dividing weight (in kilograms) by the square of the height (in meters). Waist-to-hip and waist-to-height ratios, expressed as percentages, were determined by dividing waist circumference, respectively, by hip circumference or body height.

Clinical characteristics including sitting blood pressure, total cholesterol, HDL-C, triglyceride, and history of hypertension were also recorded. Blood samples were collected from each subject after a minimum eight-hour fasting period. Total cholesterol, HDL-C, triglyceride, and glucose were analyzed using an auto-analyzer (Sysmex XE-2100 Blood Cell Analyzer, Kobe, Japan).

Central obesity was defined as a waist circumference ≥ 90 cm in males and ≥ 80 cm in females. Body adiposity index (BAI) was calculated according to the method of Bergman and colleagues, where BAI = ((hip circumference [cm])/((height [m])^1.5^) − 18) [17]. The LAP was calculated as (waist circumference [cm] − 65) × (triglyceride concentration [mM]) for men, and (waist circumference [cm] − 58) × (triglyceride concentration [mM]) for women [11]. The formula includes the minimum sex-specific waist circumference values of 65 and 58 cm, for men and women, respectively [9]. In our sample, the minimum waist circumference values for men (62 cm) and women (61 cm) were approximately 5% different to those used in the original equation for the definition of LAP. MS-TW was defined in this study according to MS criteria for Taiwanese people [18].

### Statistical analyses

Statistical analysis was performed using R software, version 2.12.1 (Free Software Foundation, Inc., Boston, MA, U.S.A.). A two-sided *P* value ≤ 0.05 was considered statistically significant. Summary data are represented as mean ± SD for continuous variables, and frequency and percentage for categorical variables. Simple logistic regression analysis was performed to test associations between MS-TW and various adiposity measures including LAP, waist-to-height ratio, BMI, waist circumference, central obesity, waist-to-hip ratio, and body adiposity index. A ROC analysis was conducted for each adiposity measures to evaluate their ability to correctly discriminate MS-TW. Plots of the sensitivity (true positive) versus 1 − specificity (false positive) were made and the overall diagnostic accuracy was quantified using AUCs. Values for each AUC can be between 0 and 1, with values greater than 0.5 desirable. A value of 1 signifies perfect diagnostic accuracy. A parameter possesses accurate diagnostic sensibility when the AUC value is greater than 0.75 [19].

AUCs of the adiposity measures were ranked in decreasing order of their values and then the adiposity measure that exhibited the greatest AUC value was compared with the next highest AUC value, and so on. Comparisons were made by applying the ROC test of DeLong using the roc.test function of the pROC library in R. Sensitivity, specificity, along with positive and negative predictive values were also estimated. The optimal cut-off was calculated as the minimum value of the square root of [(1 − sensitivity)^2^ + (1 − specificity)^2^[20], using the pROC coords function with the “closest.topleft” option selected.

## Results

Basic characteristics for the 513 study subjects are presented in Table 1. The mean age of subjects was 59.1 ± 7.0 years. Around 20.5% of subjects were classified as having MS according to MS-TW criteria, with no significant differences in the prevalence between males and females.

**Table 1:** **Basic characteristics of study subjects (*n*** **= 513)**

**Variable**	**Mean**	**Male (*n*** **= 266)**	**Female (*n*** **= 247)**	***P***
Age (years)	59.1 ± 7.0	59.3 ± 6.9*	58.8 ± 7.0	0.226
Hypertension, *n* (%)	146 (28.5)	85 (32.0)	61(24.7)	0.069
Diabetes, *n* (%)	49 (9.6)	28 (10.5)	21 (8.5)	0.436
Waist circumference (cm)	80.0 ± 8.6	84.0 ± 7.6	75.6 ± 7.3	< 0.001
Central obesity, *n* (%)	114 (22.2)	50 (18.8)	64 (25.9)	0.053
Hip circumference (cm)	92.5 ± 5.4	92.4 ± 5.3	92.6 ± 5.4	0.692
Waist-to-hip ratio (%)	86.4 ± 7.2	91.7 ± 5.4	81.7 ± 5.9	< 0.001
WHtR (%)	49.8 ± 4.9	50.6 ± 4.5	48.9 ± 5.1	< 0.001
BMI (kg/m^2^)	24.2 ± 3.0	24.5 ± 2.7	23.9 ± 3.0	0.005
Body adiposity index	27.6 ± 3.9	25.2 ± 2.7	30.1 ± 3.4	< 0.001
Systolic blood pressure (mmHg)	130 ± 19	128 ± 18	132 ± 20	0.007
Diastolic blood pressure (mmHg)	76 ± 12	79 ± 11	72 ± 12	< 0.001
Total cholesterol (mM)	5.09 ± 0.92	4.97 ± 0.96	5.21 ± 0.85	0.001
Total cholesterol ≥ 6.22 mM, *n* (%)	52 (10.1)	21 (7.9)	31 (12.6)	0.081
HDL-C (mM)	1.40 ± 0.39	1.28 ± 0.36	1.52 ± 0.39	< 0.001
HDL-C < 0.9 mM in men or < 1.03 mM in women, *n* (%)	128 (25.0)	54 (20.3)	74 (30.0)	0.012
Triglyceride (mM)	1.33 ± 0.78	1.42 ± 0.85	1.22 ± 0.70	0.002
Triglyceride ≥ 1.70 mM, *n* (%)	121 (23.6)	70 (26.3)	51 (20.6)	0.131
Fasting glucose (mM)	5.25 ± 1.08	5.29 ± 1.10	5.19 ± 1.05	0.127
Fasting glucose ≥ 5.6 mM, *n* (%)	95 (18.5)	56 (21.1)	39 (15.8)	0.125
MS-TW, *n* (%)	105 (20.5)	52 (19.5)	53 (21.5)	0.592
LAP	25.9 ± 21.6	23.0 ± 23.2	28.6 ± 19.3	0.001

*Values are mean ± standard deviation except otherwise denoted.

Central obesity: waist circumference ≥ 90 cm in males and ≥ 80 cm in females.

Body adiposity index: (hip circumference/height^1.5^) − 18.

Hypertension: subjects with a history of hypertension or using antihypertensive medications.

*HDL-C* high-density lipoprotein cholesterol, *MS-TW*: metabolic syndrome criteria for Taiwanese, *WHtR* waist-to-height ratio, *BMI* body mass index, *LAP* lipid accumulation product.

Male subjects had significantly greater waist circumferences, waist-to-hip and waist-to-height ratios, BMIs, diastolic blood pressure, and triglyceride levels compared with female subjects. However, female subjects had significantly higher body adiposity indices, systolic blood pressure, total cholesterol, HDL-C levels, and LAP compared with male subjects.

**Figure 1:**
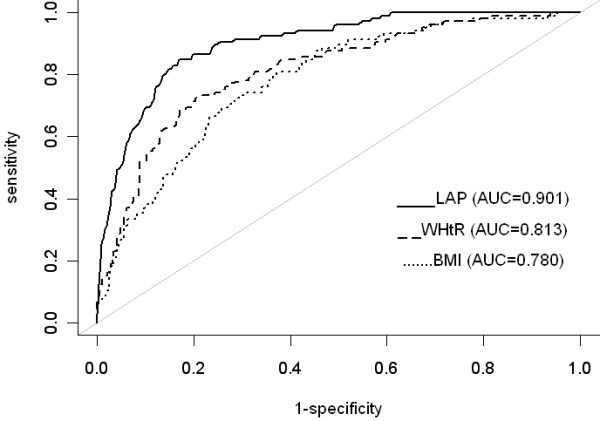
**ROC curves for adiposity measures in predicting MS using Taiwanese criteria (MS-TW). **ROC, receiver-operating characteristic; AUC, area under the curve; LAP, lipid accumulation product; WHtR, waist-to-height ratio; BMI, body mass index; MS, metabolic syndrome.

Simple logistic regression analysis indicated that all adiposity measures were significantly associated with MS-TW (Table 2). Regarding the diagnostic accuracy for MS-TW, LAP showed the highest AUC value (0.90; 95% CI, 0.87–0.93), followed by waist-to-height ratio (0.81; 95% CI, 0.77–0.86). The AUC for LAP was significantly higher than that of the waist-to-height ratio (*P* < 0.001). The AUC corresponding to waist-to-height ratio was significantly higher than that for BMI (*P* = 0.040). There were no significant differences between the remaining adiposity measures and AUCs. ROC curves with the three highest AUC values were plotted and presented in Figure 1. The codes for calculating the probability of MS in R, Microsoft Excel, or OpenOffice Calc are provided in Additional File 1.

**Table 2:** Simple logistic regression and AUC values for adiposity measures of MS using Taiwanese criteria (MS-TW)

**Adiposity measure**	**Odds ratio (95% CI)**			**Area under curve (95% CI)**		
	**Total**	**Male**	**Female**	**Total**	**Male**	**Female**
LAP	1.09 (1.08–1.12)	1.10 (1.07–1.13)	1.12 (1.08–1.15)	0.901^a^ (0.870–0.932)	0.916^c^ (0.880–0.953)	0.901^d^ (0.855–0.946)
WHtR (%)	1.32 (1.24–1.41)	1.40 (1.27–1.56)	1.28 (1.19–1.40)	0.813^b^ (0.767–0.860)	0.827 (0.762–0.892)	0.819 (0.755–0.883)
BMI (kg/m^2^)	1.45 (1.33–1.59)	1.49 (1.31–1.72)	1.43 (1.27–1.63)	0.780 (0.733–0.827)	0.776 (0.709–0.844)	0.793 (0.726–0.859)
Waist circumference (cm)	1.14 (1.10–1.17)	1.21 (1.15–1.29)	1.20 (1.14–1.27)	0.766 (0.715–0.816)	0.825 (0.760–0.890)	0.817 (0.755–0.879)
Central obesity	11.90 (7.32–19.69)	17.42 (8.48–37.32)	8.68 (4.47–17.32)	0.749 (0.700–0.799)	0.766 (0.696–0.835)	0.731 (0.661–0.802)
BMI **≥** 25 (kg/m^2^)	6.60 (4.13–10.79)	5.79 (2.98–11.90)	8.12 (4.20–16.36)	0.720 (0.672–0.768)	0.704 (0.637–0.772)	0.738 (0.669–0.807)
Waist-to-hip ratio (%)	1.12 (1.09–1.16)	1.27 (1.18–1.37)	1.20 (1.13–1.28)	0.699 (0.643–0.754)	0.784 (0.715–0.852)	0.784 (0.720–0.849)
Body adiposity index	1.16 (1.10–1.23)	1.30 (1.16–1.48)	1.24 (1.13–1.37)	0.670 (0.614–0.725)	0.726 (0.653–0.799)	0.715 (0.637–0.794)

*LAP* lipid accumulation product, *WHtR* waist-to-height ratio, *BMI* body mass index, *AUC* area under the curve.

Central obesity: waist circumference ≥ 90 cm in males and ≥ 80 cm in females.

Body adiposity index: (hip circumference/height^1.5^) − 18.

Odds ratios for all adiposity measures were significant at *P* < 0.001.

^a^*P* < 0.001 between the AUC value for LAP and that of the WHtR.

^b^*P* = 0.040 between the AUC value for WHtR and that of BMI.

^c^*P* = 0.004 between the AUC value for LAP and that of the WHtR.

^d^*P* = 0.004 between the AUC value for LAP and that of the WHtR.

Because there were significant differences in LAP between sexes, logistic regression and ROC curve analyses were conducted separately for males and females (Table 2). The greatest AUC value for LAP was 0.92 (95% CI, 0.88–0.95) and 0.90 (95% CI, 0.86–0.95) in males and females, respectively. There were no significant differences between all comparisons of AUCs for adiposity measures in either sex except that the AUC for LAP was significantly higher than that for waist-to-height ratio in both males (*P* = 0.004) and females (*P* = 0.003).

ROC curve analyses revealed that the optimal cut-off value for LAP was 28.4 with a sensitivity of 85% (95% CI, 76–91%), a specificity of 83% (95% CI, 79–87%), a positive predictive value (PPV) of 57% (95% CI, 49–65%), a negative predictive value (NPV) of 96% (95% CI, 93–97%), and an AUC value for the prediction of MS of 0.84.

In males, the optimal cut-off value for LAP was 31.6 with sensitivity of 88% (95% CI, 77–96%), specificity of 82% (95% CI, 76–87%), PPV of 55% (95% CI, 44–66%), NPV of 97% (95% CI, 93–99%) and an AUC of 0.84 for the prediction of MS. In females, the optimal cut-off value for LAP was also 31.6, with a sensitivity of 66% (95% CI, 52–78%), specificity of 93% (95% CI, 88–96%), PPV of 71% (95% CI, 57–83%), NPV of 91% (95% CI, 86–95%) and an AUC value of 0.87 for the prediction of MS.

## Discussion

We are the first to report that LAP has strong predictive accuracy, with an AUC of 0.90, for diagnosing MS in Taiwanese people. LAP is a simple indicator that requires only the determination of circulating triglycerides and measurement of waist circumference. Although waist-to-height ratio requires only a single anthropometric measurement of waist and height, the use of LAP as a predictor of MS is more advantageous. Waist circumference is unable to distinguish between visceral adipose tissue and subcutaneous adipose tissue. Visceral adiposity is more strongly associated with cardiometabolic risks compared with subcutaneous adipose tissue [21]. Visceral adipose tissue adipocytes have a higher rate of lipolysis and also produce more adipocytokines, such as interleukin-6 and plasminogen activator inhibitor-1 [22]. Therefore, it is important to include a routinely applicable indicator for evaluation of visceral adiposity. Triglyceride has been reported as a significant correlate of visceral adipose tissue in healthy men, even after controlling for abdominal subcutaneous adipose tissue [23]. Furthermore, the use of triglyceride levels in combination with waist circumference, termed hypertriglyceridemic waist, has been shown to be able to identify individuals with the greatest amount of visceral fat [24] and to be associated with increased risk of MS [25], diabetes [26], and coronary artery disease [27].

Our AUC results for LAP in MS were similar to those previously reported, and indicate the usefulness of LAP across different ethnic groups. The AUC for LAP in MS was 0.91 in a cross-sectional study of 552 healthy Argentinian men [15]. MS was defined using the revised diagnostic criteria of NCEP ATP III. Another study on 768 healthy Spanish adults also showed that LAP has the highest diagnostic accuracy for MS defined using NCEP ATP III and International Diabetes Federation criteria [28]. Moreover, a study of 40 Nigerian geriatric males reported an AUC of 0.937 for LAP in predicting MS [29].

There are some potential limitations regarding the interpretation of our results. First, a cross-sectional design was used. Future prospective studies examining MS should consider incorporating the AUC value for LAP to evaluate its utility in predicting MS and risks of cardiovascular diseases and diabetes. Second, the study results are applicable only to Taiwanese people 50 years and over. Third, individual lifestyle information, such as smoking and alcohol use, was not ascertained. These may potentially impact upon the association between obesity and MS.

## Conclusions

LAP was found to be an accurate and simple method for predicting the risk of MS in Taiwanese people, and could be effectively used by clinicians. This simple clinical tool may help, in a primary care setting, to identify subjects who require further biochemical evaluation.

## Abbreviations

LAP: Lipid accumulation product; MS: Metabolic syndrome; MS-TW: Metabolic syndrome criteria for Taiwanese; ROC: Receiver-operating characteristic; AUC: Area under the curve; BMI: Body mass index; HDL-C: High-density lipoprotein cholesterol; WHtR: Waist-to-height ratio.

## Competing interests

The authors declare that they have no competing interests.

## Authors’ contributions

J. K. C. obtained funding; contributed to the conception and design of the study; acquired, analyzed, and interpreted data; wrote and approved the manuscript. M. K. analyzed and interpreted data; and wrote, critically revised, and approved the manuscript. All authors read and approved the final manuscript.

## Pre-publication history

The pre-publication history for this paper can be accessed here:

http://www.biomedcentral.com/1471-2261/12/78/prepub

## Supplementary Material

Additional file 1 Programming code in R, Microsoft Excel and OpenOffice Calc for calculating the probability of metabolic syndrome based on the logistic regression model of lipid accumulation product. Click here for file
