# Supplementary material for: Lipid accumulation product: a simple and accurate index for predicting metabolic syndrome in Taiwanese people aged 50 and over
Source: BMC Cardiovasc Disord. 2012 Sep 24;12:78. doi: 10.1186/1471-2261-12-78 (PMC3506496; doi:10.1186/1471-2261-12-78)
Supplement: Additional file 1 — Programming code in R, Microsoft Excel and OpenOffice Calc for calculating the probability of metabolic syndrome based on the logistic regression model of lipid accumulation product. [file 1471-2261-12-78-S1.doc]

Appendix 1. Programming code in R, Microsoft Excel and OpenOffice Calc for calculating the probability of metabolic syndrome based on the logistic regression model of lipid accumulation product

1. In R environment:

To calculate the probability of metabolic syndrome according to the criteria for Taiwanese people (MS-TW) by lipid accumulation product (LAP) for males, substitute the values for the variables waist circumference (in cm) and triglyceride (in mM) in the following equation:

yhat <− (−4.122 # constant

+ 0.090*(waist circumference − 65)*triglyceride

)

phat <− 1/(exp(− (yhat))+1)

phat # copy these syntax and paste on the R console

For females,

yhat <− (−4.122 # constant

+ 0.090*(waist circumference − 58)*triglyceride

)

phat <− 1/(exp(− (yhat))+1)

phat # copy these syntax and paste on the R console

1. In Microsoft Excel or OpenOffice Calc:

Key in the values for waist circumference (in cm) in the A1 cell and triglyceride (in mM) in the A2 cell

For males, key in the following formula in any empty cell on the spreadsheet to obtain the probability of metabolic syndrome according to the criteria for Taiwanese people (MS-TW):

=1/EXP(− (−4.122+0.090*(A1−65)*A2)+1)

For females,

=1/EXP(− (−4.122+0.090*(A1−58)*A2)+1)

Note: The model should only be applied to individuals with triglyceride between 0.305 mM and 6.79 mM. In addition, waist circumference should not exceed 110.5 cm. When waist circumference is less than 65 cm for males and 58 cm for females, the probability of MS is < 0.006 .
